# Effects of environmental enrichment on growth performance, carcass traits, meat quality, and hair follicle development of Rex rabbits

**DOI:** 10.5713/ajas.20.0540

**Published:** 2020-10-22

**Authors:** Yang Feng, Hao Shi, Shuangbao Gun

**Affiliations:** 1 College of Animal Science and Technology, Gansu Agricultural University, Lanzhou 730070, China

**Keywords:** Carcass Trait, Environmental Enrichment, Growth Performance, Hair Follicle, Meat Quality, Rex Rabbits

## Abstract

**Objective:**

The purpose of this study was to investigate growth performance, carcass traits, meat quality and hair follicle development of growing Rex rabbits as affected by different environmental enrichment materials.

**Methods:**

A total of one hundred and twenty Rex rabbits were randomly assigned to four groups; reared in conventional cages (not enriched) and in enriched cages with either willow stick (WS), rubber duck, or a can containing beans (CB), for 44 days.

**Results:**

The average daily gain of the CB group was the highest and had a significant difference from that of the other groups (p<0.05). The spleen and cecum weight of the CB group was greater than those of the WS and control groups (p<0.05). The redness (Commission Internationale de l’Eclairage a*) of the meat sample of the control group was lower than those of the enriched cage groups (p<0.05). Moreover, the hue value of the CB group was significantly lower than that of the other groups (p<0.05). The tenderest meat belonged to the CB group. In addition, more secondary (p<0.05) and primary follicles were found in the CB group than in the control group.

**Conclusion:**

Environmental enrichment increased the average daily gain and improved some carcass traits, meat quality, and hair follicle density. Among the three environmental enrichment materials, CB could be recommended for rabbit husbandry.

## INTRODUCTION

Environmental enrichment (EE) refers to the provision of materials for the environment of captive animals that meet the specific needs of the species in order to improve its physical and psychological health [1]. The lack of EE is often considered as an animal welfare problem. The EE can relieve the pressure that comes from the environment, reduce abnormal behaviors, improve animal welfare, and enhance animal production [2,3].

The EE positively influences production in other animals species: Winfield et al [4] and Day et al [5] reported that enrichment materials can be utilized to reduce the occurrence of abnormal behaviors in pigs (e.g., tail biting, ear biting), and improve their meat quality and survival rates. Also, various studies on blue foxes, mice, guinea pigs, chickens, and sheep [2,6,7] have been demonstrated, and the results show that EE has a positive effect on animal behavior or production.

Similarly, EE plays an important role in rabbit production. In recent studies, researchers attempted to improve the production performance of rabbits using EE. They found that an enriched cage may positively affect the welfare, prevent abnormal behaviors or stereotypes (such as cage bar biting and conus circling), reduce anxiety, and improve the growth performance and carcass traits of rabbits [8,9]. However, in previous studies, Bozicovich et al [3,10,11] often used a particular kind of EE or compared the same type of EE materials, such as apple stick vs. willow stick. Minimal research has been conducted on the comparison of EE with totally different materials and properties on rabbits, thus it is difficult to summarize which EE material is more effective for them. The summarization is required to ascertain the best EE for rabbits, which is done by comparing the effects of different types of EE materials on animal production.

The EE materials should stimulate the visual, somatosensory, and olfactory systems of animals while providing an aspect of novelty [12]. Benaroya-Milshtein et al [2,5,6] have found that straw, wood chips and bark, toy balls, colorful plastic bottles, and even newspapers, could improve the welfare of animals, such as mice, pig, and lamb, and improve their survival rate and meat quality. However, for rabbit, recent studies about enrichment materials involved only certain types of wooden sticks, hay, grasses, or straws [8,9]. Hence, the optimal EE materials for the welfare of rabbit remains unclear. Although rabbits have adapted to captivity, their behavior and physiology are still similar to those of their wild counterparts, as such, exposing them to certain benefits such as exploration, foraging, and gnawing are necessary for them to meet their physiological and behavioral needs [1]. Therefore, we hypothesized that rabbits might be more interested in soft and flexible enrichment materials than wooden stick. In the present study, we chose three types of enrichment materials (willow stick [WS], rubber duck [RD], and can containing beans [CB]) that could be gnawed and played with, to reduce the stress resulting from the lack of stimuli and improve rabbit welfare. A WS is a common EE for rabbits, while RD is a colorful, soft, and easy to gnaw material that can be hung in the cages; consequently, these materials can satisfy the exploration need of rabbits. Moreover, the curiosity of rabbit is satisfied from the noise emanating from CB when it is being moved from one place to another by the rabbit. The RD and CB were the EE materials chosen to encouraging gnawing and playing according to the biological characteristics of the rabbit. We compared them with WS, a traditional EE material most often used in rabbit husbandry, and conventional cages (not enriched). These three kinds of EE materials are all totally different types that were chosen to satisfy the different needs of rabbits. The aim of the study was to identify the most suitable EE material for rabbits.

In this study, we measured the growth performance, carcass trait, and meat quality of growing rabbits in different enriched cages. Furthermore, besides high-quality protein meat, Rex rabbits are also widely known for Rex fur, which is soft, dense, and of uniform length [13]. Some studies have shown that emotion (bad mood as a result of an illness or inadequate feeling) may have a negative effect on hair growth, and stress can cause hair loss [14,15]. EE is known to reduce stress, and at present, little data is available on the effect of EE on hair follicle (HF) development. The primary follicle (PF) and secondary follicle (SF) density could reflect the quality of HF development. Therefore, we hypothesized that EE could have a positive effect on the PF and SF density.

This study was conducted to investigate the influence of the chosen EE on growth performance, carcass traits, meat quality and HF density of Rex rabbits. Among the three different types of EE materials, we explored the best one for rabbits and suggested it for the rabbit farm.

## MATERIALS AND METHODS

The experiment was conducted on July 8–August 23, 2019, at the YuanFa Rex Rabbit Farm located in Baiyin, Gansu Province, China. All live rabbits procedures were approved by the Gansu Agricultural University Animal Care and Use Committee (Approved no. 2019-2-142). All animal care and use were consistent with the Regulations for the Administration of Affairs Concerning Experimental Animals (The State Science and Technology Commission of P.R. China, 1988).

### Animal feeding and housing

One hundred and twenty 2-month-old Rex rabbits (60 males and 60 females) were randomly divided into four groups (n = 30, half male and half female), with two rabbits as replicate. The rabbits were housed in pairs in a wire cage measuring 60 cm×45 cm×40 cm with bamboo flooring. The rabbits were raised in a temperature range of 17°C to 24°C and under natural light conditions. They were fed pelleted diet *ad libitum* which 4 mm diameter, and water was available on demand from nipple drinkers. Fattening began at the age of two months, and they were reared for 44 days. During the experimental period, which lasted for 60 to 105 days of age, rabbits were inspected daily; no health problems were observed. The diet formula was prepared according to the dietary requirements by the Nutritional Research Council for rabbits (Table 1).

### Environmental enrichment

**Figure 1:**
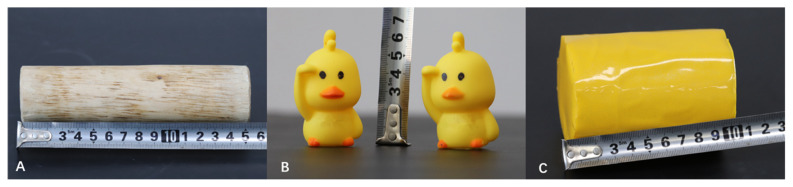
Environmental enrichment materials. (A) Willow stick; (B) Rubber duck; (C) Can containing beans.

Before the start of the study, the rabbits had been housed for ten days without the provision of enrichment material. When the experiment started, three types of EE materials (WS, RD, and CB) were used in the enriched treatment groups (Figure 1). The RDs (Figure 1B) were hung at approximately 20 cm from the bottom of the cages, while the WSs (Figure 1A) and CBs (Figure 1C) were placed at the base of the cage. The beans content of the can made series of sounds when touched by the rabbit.

### Growth performance

The body weights were measured every week. Feed intake (FI) was recorded throughout the experiment. The feed conversion ratio (FCR) and average daily gain (ADG) were calculated according to the procedure described by Jordan [8]. The methods for measuring body length, chest depth, chest circumference (heart girth), dorsum length (from the cervical part to the base of the tail), hind leg, and rump circumference were based on those proposed by Mcnitt et al [13,16]. The body index and chest depth index were calculated using the following equations:

$$\begin{array}{l} \text{Body index}=\text{chest circumference}/\text{body length}\\ \text{Chest depth index}=\text{chest depth}/\text{body length.} \end{array}$$

### Carcass traits and meat quality

Feed and water were withdrawn for 24 h before slaughter. The rabbits were stunned, slaughtered, immediately eviscerated, and then skinned. The carcass (without the head, legs, tail, viscera, fat deposits and blood), skin, liver, heart, kidney, and spleen were weighed. Dressing out percentage was calculated as follows:

Dressing out %=(carcass weight/slaughter weight)×100.

The contents of both the stomach and cecum were removed before weighing. The skin area (cm^2^) was determined using the following equation:

$$\text{Skin area }({\text{cm}}^{2})=\text{skin length }(\text{cm})\times \text{skin width }(\text{cm}).$$

Lightness (L*), redness (a*), and yellowness (b*) values were measured in the left thigh muscles and buttock parts within 40 min post-mortem using a chromameter (Minolta Chroma Meter, Hamburg, Germany) operating under D65 illuminant and 10° observer angle. Each part was measured three times, and the average values were calculated [17]. The Commission Internationale de l’Eclairage (CIE) L* refers to the measure of lightness, the larger the number, the lighter the color. CIE a* indicates a red (+) to green (−) color scale, and CIE b* denotes the yellow (+) to blue (−) color scale. The hue angle (Hue) which defines color (0°-red; 90°-yellow), was calculated as an arctangent (b*/a*). Chroma denotes the color intensity (0-dull; 60-vivid), and computed as follow:

$$\text{Chroma}=\sqrt{{\text{a}}^{*2}+{\text{b}}^{*2}}$$[
18].

Meat tenderness value was measured from the hind leg using a meat tenderness meter (TENOVO C-LM3, Beijing, China). The pH value of the hind leg was determined using a pH meter (SuYuan PHS-3C, Shanghai, China) within 45 min post-mortem.

### Skin collection and hair follicles assays

Skin samples measuring 2 cm in diameter, were collected from the center of the buttocks of the experimental rabbits. After rinsing with normal saline, the samples were immediately soaked in a paraformaldehyde fixative solution.

The samples were dehydrated with alcohol gradient, embedded in paraffin, and sliced in 6 μm sections after 48 h, stained with hematoxylin and eosin and observed under a digital microscope (Moti, BA210, Xiamen, China). Seven images (magnifcation of 40×) of different view were obtained for each section, and the HF diameter and density were examined using the Motic Images Plus 2.0 system. The diameters of the PF and SF were measured, and the number of PFs and SFs were counted in each image.

### Statistical analysis

Data were analyzed using the SPSS 20 software. Seven images were obtained for hair follicle numbers in each section. They were obtained repeatedly and analyzed by repeated measures analysis of variance (ANOVA). Other data were evaluated by one-way ANOVA. In the study, the Turkey's test was used to determine the significant difference, and p<0.05 was defined as significant.

## RESULTS

### Growth performance

The four experimental groups produced no mortality. The slaughter weights (final live body weight) of the enriched cage groups were higher than those of the control group. The ADG of the CB group was the highest at 25.63 g, significantly higher than that of the control group (p<0.05), and the ADG of the enriched groups was also higher than that of the control group. The lowest FCR was observed in the CB group, and the WS and RD groups had lower FCR values than the control group. The body length of the control group was higher than that of the WS group. The chest circumference of the CB group was the highest. The highest body index (77.22%) was also found in the CB group. The chest depth index, rear leg and rump circumference, and body index of the control group were lower than those of the enriched cage groups (Table 2).

### Carcass trait

The carcass weight and dressing out percentage of the control group were the lowest (1,414 g and 54.93%, respectively). The skin area of the CB group was the largest, while the WS and RD group had greater skin area than the control group. The stomach weight of the control group was the greatest and was significantly greater than those of the WS and RD groups (p<0.05). The spleen weight of the CB group was significantly greater than those of the other groups (p<0.05). The cecum weight of the CB group was significantly greater than those of the WS and control groups (p<0.05) (Table 3). The EE exerted no significant effect on the liver, heart, and kidney weights and the small intestine lengths of the four groups.

### Meat quality

CIE L* and CIE a* of the control group were the lowest and the CIE a* of the RD group was the highest (p<0.05).The Hue value of the RD group was significantly lower compare to the rabbits raised without EE (Hue = 2.42, p<0.05). The meat sample of the CB group was the most tender (5.72 N, p<0.05). No significant effect was found in the other measured parameters (Table 4).

### Hair follicle

The PF diameters (p<0.05) and SF diameters (p<0.05) of the WS and CB groups were lower than those of the control and RD groups. More SFs (p<0.05) and PFs (p<0.05) were also found in the CB group than in the other groups (Table 5).

## DISCUSSION

### Growth performance

The rabbits in the enriched cages had higher slaughter weights and FI than those of the control group (non-significant). The slaughter weight results were in agreement with those found by Maertens et al [19], Luzi et al [9], Princz et al [20], and Mohammed and Nasir [11], who reported that EE exerts a positive effects on the weight gain of rabbits. However, the results of the current work are contrary to those obtained by Jordan et al [8–10,21] who reported that EE did not have effect on the slaughter weight. They used gnawing sticks, wood, bamboo as EE for fattened or weaned rabbits. Such discrepancy may be attributed to the different enrichment materials and growth periods of rabbits. The enriched cage groups had lower FCR, and higher ADG (p<0.05) than those of the control group, and this supports the findings of Mohammed and Nasir [11]. This result could be attributed to the amount of time rabbits spend playing and gnawing. Gnawing is associated with digestive mechanisms and the nervous system, which could decrease stress and promote intestinal flow, digestion and overall animal health [21,22]. Decreasing stress might stimulate the appetite, increase the digestibility of dietary components, enhance the digestion of dry matter and energy release, influence hormones and the enzymes released, and consequently, improve the process of digestion [21,23]. In this study, the CB group had the highest ADG and the lowest FCR among all groups. This result may be attributed to the fact that the appropriate sound of cans could decrease stress by inducing playing behavior [24]. The CB exerted a considerable impact on the productive performance of the rabbits. In this study, the FCR in the RD group was higher than those in the other groups. This result may be attributed to the fact that the RD is yellow and soft and hangs in the cage. The rabbits are sensitive to the yellow color [25], and soft materials are easy for gnawing. The RD hangs in the cage, and sway when touched it. These characteristics made this enrichment material attractive to the rabbits and led to increased gnawing and playing, relative to the other groups. None of the enrichment materials showed harmful effects on the rabbits during the entire experiment.

### Carcass trait

In accordance with other studies involving the use of EE for rabbits, similar carcass trait performances with and without enrichment were obtained [9,20]. The carcass weight was higher in the enriched cage groups than in the control group. The RD group showed the highest carcass weight, which may be due to their heavier slaughter weights. The dressing-out percentage in this study was numerically higher (non-significant) in the enrichment groups than in the control group; this outcome is in agreement with the results of Kermauner et al [21] used wooden sticks for cage enrichment. The largest skin area was observed in the CB group, possibly because the chest circumference and chest depth of the CB group were larger than those in the other groups. The fur value of Rex rabbits depends on their hair quality and skin area. A large skin area is deemed highly valuable. Cage enrichment could increase the skin area, and this result may be related to the higher slaughter weight.

In this study, no significant difference was observed in the other carcass traits (the heart, liver, kidney, and small intestine) of the groups with and without EE. This outcome is compatible with the results of Kermauner et al [21], Princz et al [10], Jordan et al [8], and Mohammed and Nasir [11]. The spleen and cecum weight of the CB group was significantly greater than that of the control group (p<0.05). There are no previous reports on the effect of EE on the spleen; therefore, we currently have no reasonable explanation for this result. Rabbits are true non-ruminant herbivores with a particular type of digestion called cecum fermentation [26]. The cecum is an important and large digestive organ in rabbits and is assumed to have a similar function as the rumen in cattle. It contains a rich population of bacterial that could aid digestion through fermentation and the synthesis of B vitamins. Additionally, the soft feces of rabbits are produced in the cecum, that is significantly for rabbits’ digestion. The weight of a rabbit’s cecum can reflect its developmental condition. In the current study, the daily gain of the CB group was the highest, and the FCR was the lowest, meaning that the rabbits in the CB group had better feed utilization, possibly because of the well-developed cecum.

### Meat quality

Cage enrichment did not influence the pH, CIE L*, CIE b*, and Chroma; however, it increased the CIE a* value and decreased the hue value. Meat redness mainly depends on the activities of rabbits, with more activity resulting in a darker muscle color [27]. In this study, the rabbits in the enriched cages played with the enrichment materials and showed much activity. The RD is colorful, soft, and hangs in the cage, these characteristics made it attractive to the rabbits and led to increased gnawing and playing, relative to the other groups. This may explain the dark meat color of the enriched groups. It is in agreement with the result of Jordan et al [8], who added wooden sticks to rabbit cages. In contrast, Luzi et al [9] and Kermauner et al [21] registered higher CIE a* in animals without wooden stick. However, they did not consider the hue; therefore, they could not determine whether or not the meat of rabbits in conventional cages (without enrichment) is redder than that of rabbits in enriched cages. In our study, the hue value of the RD group was significantly lower than that of the control group (2.42, p<0.05), indicating that the meat color of the CB group was the best. Furthermore, the meat of the CB group showed the most tenderness. Therefore, improving meat quality by increasing the richness of the caged environment is beneficial. Overall, CB had a great positive effect on the meat quality of rabbits.

### Hair follicles

The fur quality of Rex rabbits depends largely on hair density [28]. Stress may cause hair loss and inhibit hair growth by activating the substance P dependence of macrophages or mast cells in the context of brain-hair follicle axis [29], which indicates that moods can affect hair growth. In this study, we found that the SF population of the CB group was greater than those of the other groups. Throughout the experiment, the rabbits in the CB group spent most of their time playing with the sound-producing cans. A common effect of EE is to reduce stress, therefore, this result indicates that CBs might have contributed to providing a less stressful environment and exerted a positive effect on the SF growth. Allison et al [30] reported that providing an EE material to C57BL/6J mice can delay the onset of alopecia and reduce its prevalence and overall severity. This result is similar to our findings and may indicate that husbandry methods promote the well-being of the animals and also improve their hair density. In this study, the PF density did not changed possibly because no additional PFs are formed after birth, although the size of hair follicles and strands can change slightly over time under the influence of androgens [28]. Intervention in postnatal mammalian HF had a relatively significant effect on SF only, thus in the present study, we only found that EE could affect the SF density. SF is an important micro-organ that grows the hair of underfur, therefore, the SF density could affect the underfur density and an improved SF density has a great effect on fur quality. From the study, we predicted that EE could be used to increase the SF density in Rex rabbits and among the three experimental EE materials we recommend the use of CB because it had the highest positive effect on the SF density.

## CONCLUSION

In this study, cage enrichment increased the ADG, spleen, and cecum weight of Rex rabbits. It also improved meat tenderness and meat color, and HF density. These traits are all important for Rex rabbit husbandry. Our results support that EE can reduce the stress of captive rabbits which has a positive effect on these traits. However, in the present study, we did not measure other functions, such as whether enriched materials could improve the physiological and immune functions of rabbits, and this warrants further research. Among the three types of EE materials tested, CB was found to be the most beneficial for rabbit production; therefore, it can be used for rabbit husbandry.

## Figures and Tables

**Table 1:** Ingredients and chemical composition of the basal diet

Items	Level
Ingredient (%, air dry basis)	
Alfalfa meal	62.80
Corn	15.99
Wheat bran	6.11
Soybean meal	6.51
Greaves	4.08
Premix^1)^	4.00
Salt	0.32
Sodium bicarbonate	0.09
Allicin	0.10
Chemical composition of concentration (DM basis)	
DE (MJ/kg)	10.35
CP (%)	17.52
CF (%)	15.56
Calcium (%)	0.83
Phosphorus (%)	0.41

DE, digestible energy; CP, crude protein; CF, crude fiber; Ca, calcium; P, phosphorus.

1) Premix provided the following per kg of the diet: vitamin A, 8,000 IU; vitamin B_1_, 1.8 mg; vitamin B_2_, 6 mg; vitamin B_6_, 0.3 mg; vitamin D, 800 IU; vitamin E, 50 mg; Cu, 50mg; Fe, 100 mg; Zn 50 mg; Mn 30mg; Mg 150 mg; Se 0.1 mg.

2) CP, CF, Calcium, and phosphorus were measured values, while DE were calculated values.

**Table 2:** Growth performance of rabbits housed in enriched cages with WS, RD, CB and conventional cages

Trait	Control group	Cage enrichment group^1)^			SEM	p-value

		WS group	RD group	CB group		
Initial body weight (g)	1,926.67	1,890.00	1,993.08	1,896.00	36.17	0.360
Slaughter weight (g)	2,544.00	2,635.71	2,668.46	2,659.33	57.33	0.451
Average daily gain (g)	17.39^b^	21.15^b^	19.12^b^	25.63^a^	1.91	0.035
Daily feed intake (g)	150.85	157.20	149.69	156.50	6.91	0.770
Feed-conversion ratio	9.06	7.60	8.05	7.43	0.46	0.129
Body length (cm)	42.67	41.13	42.00	41.87	0.49	0.143
Chest circumference (cm)	30.73	30.63	31.87	32.30	0.51	0.117
Dorsum length (cm)	33.60	32.27	33.60	33.07	0.52	0.289
Chest depth (cm)	9.35	9.15	10.08	9.7	0.36	0.301
Rear leg and rump circumference (cm)	30.97	32.47	33.20	31.80	0.60	0.053
Body index (%)	72.12	74.64	75.95	77.22	1.34	0.191
Chest depth index (%)	21.94	22.39	23.98	23.18	0.88	0.409
No of replication	15	15	15	15	-	-

SEM, standard error of the mean.

1) WS group, willow stick group; RD group, rubber duck group; CB group, can containing beans group.

a,b Different letters on the same rows mean signifcant difference at p<0.05 level.

**Table 3:** Carcass traits of rabbits housed in enriched cages with WS, RD, CB and conventional cages

Trait	Control group	Cage enrichment group^1)^			SEM	p-value

		WS group	RD group	CB group		
Carcass weight (g)	1,414.00	1,472.50	1,517.69	1,449.20	38.17	0.323
Dressing out (%)	54.93	56.18	56.90	55.69	1.30	0.767
Skin weight (g)	272.67	281.33	274.67	276.00	14.71	0.931
Skin area (cm^2^)	876.48	900.95	933.17	951.93	49.00	0.718
Liver (g)	94.37	91.16	96.04	88.99	5.13	0.774
Heart (g)	6.31	6.16	6.74	6.39	0.27	0.527
Small intestine length (cm)	277.60	287.13	293.07	268.70	13.00	0.609
Kidney (g)	16.18	15.10	16.45	15.42	0.82	0.623
Empty stomach (g)	25.18^a^	21.64^b^	21.45^b^	23.63^ab^	1.02	0.041
Spleen (g)	1.36^b^	1.34^b^	1.32^b^	1.89^a^	0.14	0.019
Empty cecum (g)	21.91^b^	21.79^b^	26.32^ab^	29.63^a^	2.34	0.043
No of replication	15	15	15	15	-	-

SEM, standard error of the mean.

1) WS group, willow stick group; RD group, rubber duck group; CB group, can containing beans group.

a,b Different letters on the same rows mean significant difference at p<0.05 level.

**Table 4:** Meat quality of rabbits housed in enriched cages with WS, RD, CB and conventional cages

Items		Control group	Cage enrichment group^1)^			SEM	p-value

			WS group	RD group	CB group		
Meat colour	L*	45.40	47.29	47.42	46.90	0.74	0.208
	a*	2.02^b^	2.74^ab^	3.36^a^	2.18^ab^	0.43	0.042
	b*	7.36	9.25	7.78	6.97	0.79	0.262
	Chroma	7.60	9.77	8.52	7.64	0.82	0.288
	Hue	3.47^a^	3.15^ab^	2.42^b^	3.06^ab^	0.37	0.001
Tenderness (N)		6.95^ab^	7.21^a^	6.04^ab^	5.72^b^	0.49	0.045
pH		6.49	6.29	6.45	6.36	0.07	0.21
No of replication		15	15	15	15	-	-

SEM, standard error of the mean; L*, lightness; a*, redness; b*, yellowness.

1) WS group, willow stick group; RD group, rubber duck group; CB group, can containing beans group.

a,b Different letters on the same rows mean significant difference at p<0.05 level.

**Table 5:** Hair follicle development of rabbits housed in enriched cages with WS, RD, CB and conventional cages

Trait	Control group	Cage enrichment group^1)^			SEM	p-value

		WS group	RD group	CB group		
PF diameter (μm)	56.25^a^	47.34^b^	57.84^a^	47.17^b^	2.78	0.018
SF diameter (μm)	23.97^ab^	21.12^b^	26.77^a^	21.92^b^	1.12	0.006
PF density (per field)	27.87^ab^	26.35^b^	25.42^b^	29.50^a^	2.29	0.015
SF density (per field)	66.45^b^	79.29^b^	80.52^b^	92.50^a^	3.80	0.031
No of replication	15	15	15	15	-	-

SEM, standard error of the mean.

PF, primary follicle; SF, secondary follicle; per field means per low power filed at 40×.

1) WS group, willow stick group; RD group, rubber duck group; CB group, can containing beans group.

a,b Different letters on the same rows mean significant difference at p<0.05 level.
